# Beyond lactate: prognostic value of metabolic and biochemical parameters in methanol poisoning - a retrospective observational study

**DOI:** 10.1186/s12873-026-01492-0

**Published:** 2026-02-07

**Authors:** Ecem Ermete Güler, Ejder Saylav Bora, Mehmet Göktuğ Efgan, Serkan Bilgin

**Affiliations:** 1 Present Address: Department of Emergency Medicine, Izmir Ataturk Training and Research Hospital, Izmir, Turkey; 2 Department of Emergency Medicine, Izmir Katip Celebi University Faculty of Medicine, Izmir, Turkey

**Keywords:** Methanol poisoning, Lactate, Lactate clearance, Anion gap, Base excess, Prognosis, Emergency department

## Abstract

**Background:**

Methanol poisoning carries high mortality and morbidity, and early risk stratification in the emergency department (ED) remains challenging. We evaluated the prognostic value of lactate dynamics alongside readily available metabolic markers.

**Methods:**

This single-center, retrospective observational study included adults (≥18 years) presenting to the ED with methanol intoxication between 01/2020–03/2023. Demographics, clinical findings, and laboratory values were extracted from electronic records. Lactate was measured at 0, 2, and 4 hours; lactate clearance was calculated as (Initial lactate−Follow-up lactate)/Initial lactate × 100. Outcomes were in-hospital mortality, intensive care unit (ICU) admission, and discharge. Group comparisons and ROC analyses were performed (α = 0.05).

**Results:**

Eighty-two patients were included (mean age 52.9 ± 14.2 years; 92.7% male). In-hospital mortality was 11.0% and ICU admission 54.9%. Non-survivors had higher lactate at presentation (9.04±6.09 vs 4.21 ± 3.83 mmol/L, *p* < 0.001) and markedly lower/negative lactate clearance (−33.4±95.3% vs 43.1 ± 29.9%, *p* < 0.001). Lactate predicted mortality with AUC 0.785 (cut-off > 8.5 mmol/L; sensitivity 77.8%, specificity 72.6%). Delta lactate was associated with mortality on univariate analysis but did not yield a clinically useful cut-off. For level of care outcomes, anion gap predicted ICU admission (AUC 0.722), while anion gap ≤18.4 mmol/L predicted discharge (AUC 0.793; sensitivity 85.1%, specificity 73.3%). Base excess contributed to prediction of ICU admission and discharge.

**Conclusions:**

In methanol intoxication, both admission lactate and early lactate clearance are strong prognostic indicators of mortality. Anion gap and base excess add complementary value for predicting ICU need and discharge. Incorporating these routinely available parameters into ED assessment may improve early risk stratification and guide timely management decisions.

## Introductıon

Methanol intoxication is among the toxic alcohol emergencies with the highest rates of mortality and morbidity. Delays in diagnosis and treatment play a decisive role in survival [1]. Exposure most commonly occurs through ingestion of counterfeit alcoholic beverages, industrial solvents, windshield washer fluids, or antifreeze. Although oral intake is the predominant route, inhalation and dermal absorption may also lead to toxicity [2]. Once ingested, methanol is metabolized by alcohol dehydrogenase to formaldehyde and subsequently to formic acid. These toxic metabolites inhibit mitochondrial cytochrome c oxidase, resulting in cellular hypoxia and severe metabolic acidosis [3]. Clinically, patients may present with nausea, vomiting, visual disturbances, and altered consciousness, and without timely intervention, the course can rapidly progress to coma and death [4].

Accurate prognostication in methanol intoxication is essential for guiding treatment strategies. Lactate concentration has long been recognized as a reliable biomarker of cellular hypoxia, with levels above 4–5 mmol/L associated with markedly increased mortality risk [5, 6]. Elevated lactate is closely related to tissue hypoxia and metabolic acidosis driven by formic acid accumulation, while alterations in the NADH/NAD ratio during methanol metabolism further contribute to lactic acidosis [7]. However, single-point lactate measurements may fail to adequately capture the dynamic metabolic response. For this reason, lactate clearance has emerged as a more robust indicator of tissue perfusion and metabolic recovery. Several studies in critical illness—including sepsis, cardiac arrest, and trauma—have demonstrated that higher lactate clearance is strongly associated with survival, whereas reduced clearance reflects poor response to treatment and worse outcomes [8, 9]. Similarly, investigations in severe ischemic conditions such as mesenteric ischemia and acute coronary syndrome suggest that lactate clearance may serve as an independent prognostic marker [10, 11].

Given the high mortality and morbidity of methanol poisoning, rapid initiation of effective treatment in the emergency department is of paramount importance [12]. Yet diagnosis and assessment of severity are often challenging due to limitations such as impaired history-taking in patients with altered consciousness, difficulties in measuring serum methanol concentrations, and the lack of gas chromatography facilities in many hospitals [12–14]. Consequently, there is a need for widely accessible laboratory parameters that can support prognostic assessment across all emergency settings. In this context, lactate clearance represents not only a biochemical marker of tissue hypoxia but also a potential indicator of treatment response and survival. However, the prognostic role of lactate clearance has not been fully elucidated in the context of methanol intoxication. Most existing research has focused on ICU mortality and dialysis requirements, while no reliable biomarker has yet been established to predict hospital discharge outcomes.

The present study therefore aims to evaluate the relationship of lactate levels, lactate clearance, anion gap, base excess, and glucose-to-potassium ratio with prognosis in patients presenting to the emergency department with methanol intoxication, with the goal of providing additional evidence to guide clinical decision-making.

## Materıals and Methods

### Study design and setting

This retrospective, observational study was conducted in the Emergency Department of İzmir Katip Çelebi University Atatürk Training and Research Hospital. Ethical approval was obtained from the Non-Interventional Clinical Research Ethics Committee of İzmir Katip Çelebi University (Decision No: 0176, Date: 27.04.2023). The study included adult patients aged 18 years and older who presented to the emergency department with a diagnosis of methanol intoxication between January 1, 2020, and March 31, 2023.

### Patient selection and diagnostic approach

Adult patients (≥18 years) who presented to the emergency department with altered mental status, visual disturbances, or signs of metabolic acidosis, and who had a history of illicit/unrecorded or homemade alcohol consumption, were included in the study. “Suspected methanol ingestion” was defined as the concurrent presence of a reported history of unrecorded or homemade alcohol consumption, severe metabolic acidosis disproportionate to the clinical context, and accompanying neurological and/or visual symptoms. The diagnostic criteria included an arterial blood gas pH < 7.3, bicarbonate (HCO₃^−^) < 20 mEq/L, and an anion gap > 12 mEq/L.

**Fig. 1:**
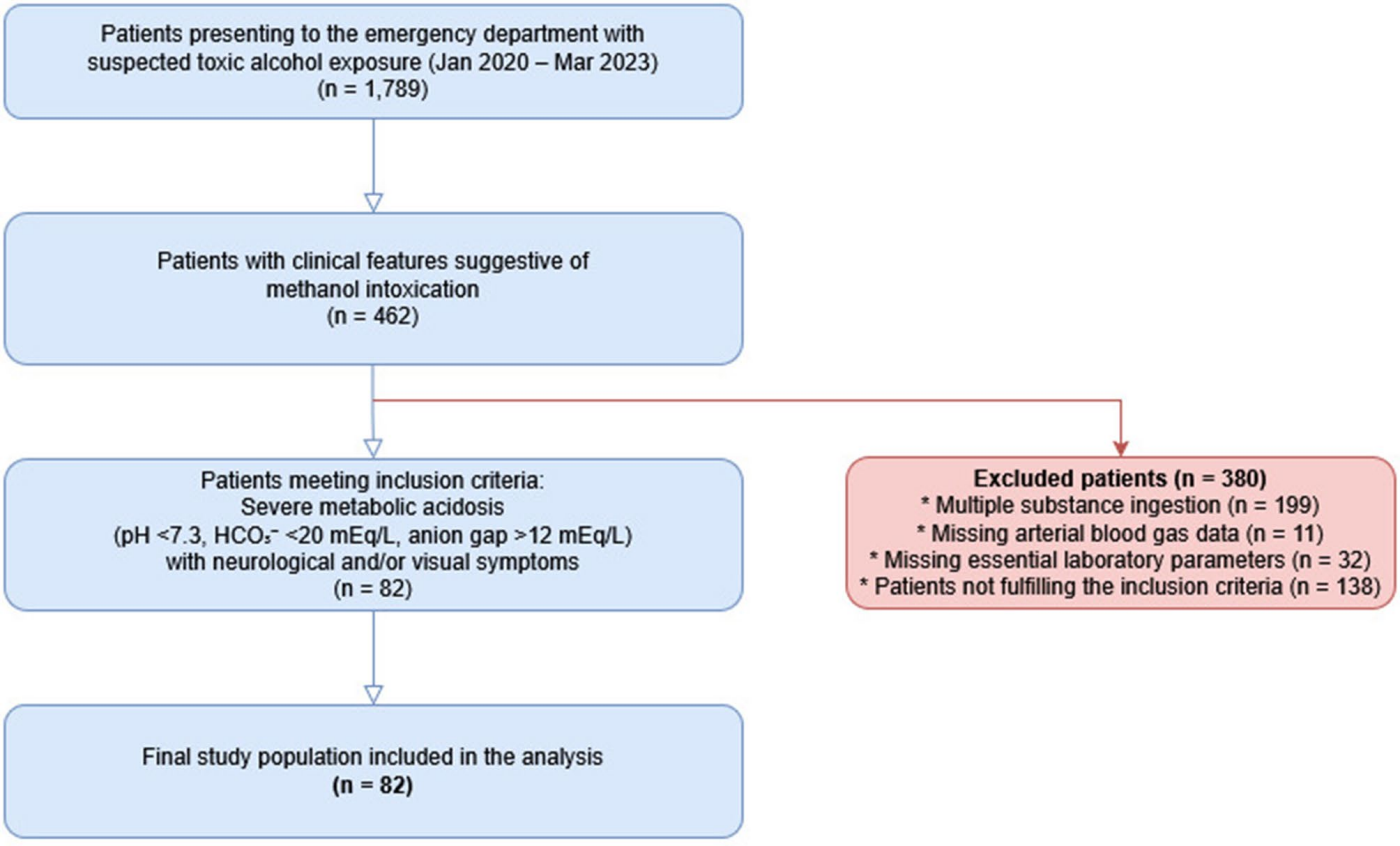
Flowchart of the study

At the study center, serum methanol or formate measurements, assessments required for osmolar gap calculation, and gas chromatography/mass spectrometry (GC/MS) analyses are not routinely available in the emergency department setting. Therefore, the diagnosis of methanol intoxication was established based on a combination of exposure history, clinical findings, and laboratory parameters. Patients with identified multiple substance ingestion or incomplete blood gas and essential laboratory data were excluded from the study (Fig. 1).

### Data collection

Patient data were obtained from the hospital information management system and electronic medical records. Recorded variables included demographic characteristics (age, sex, mode of presentation), clinical findings (level of consciousness, visual complaints, vital signs), and laboratory results (blood gas parameters: pH, HCO₃^−^, PCO₂, lactate; biochemical parameters: BUN, creatinine, sodium, potassium, chloride, calcium; serum ethanol levels). Clinical outcomes evaluated were intensive care unit (ICU) admission, discharge status, length of hospital stay, and in-hospital mortality.

### Lactate measurements and clearance calculation

Lactate levels were measured at emergency department presentation (0 hour) and during routine follow-up at approximately the 2nd and 4th hours, consistent with standard clinical monitoring of patients with severe metabolic acidosis and suspected toxic alcohol ingestion. These time points reflect real-world emergency department practice rather than protocolized research sampling. Due to the retrospective design, the exact timing and dosing of therapeutic interventions such as antidote administration, sodium bicarbonate infusion, and renal replacement therapy could not be consistently documented relative to lactate measurements. Therefore, lactate clearance was evaluated as a dynamic prognostic marker reflecting overall metabolic response during early management rather than as a direct indicator of specific treatment effects. Lactate clearance was calculated using the formula:

Lactate Clearance (%) = (Initial lactate−Follow-up lactate)/Initial lactate × 100

Positive values indicated a decrease in lactate levels and a favorable treatment response, whereas negative values reflected an increase in lactate levels and a poor clinical course.

### Statistical analysis

Statistical analyses were performed using IBM SPSS Statistics version 26.0 (IBM Corp., Armonk, NY, USA) and MedCalc (MedCalc Software Ltd., Ostend, Belgium). Descriptive statistics were presented as mean ± standard deviation, median, minimum, and maximum values. The Shapiro–Wilk test was used to assess normality, and the Levene test was applied for homogeneity of variance. Normally distributed variables were compared using the Independent Samples t-test, while non-normally distributed variables were compared using the Mann–Whitney U test. Categorical variables were analyzed using Pearson’s chi-square or Fisher’s exact test. The prognostic performance of lactate and lactate clearance was evaluated with Receiver Operating Characteristic (ROC) curve analysis, and optimal cut-off values were determined considering sensitivity and specificity. A p-value < 0.05 was considered statistically significant.

## Results

A total of 82 patients were included in the study. The mean age of the participants was 52.86 ± 14.24 years, ranging from 16 to 77 years. With respect to sex distribution, 92.7% of the patients were male and 7.3% were female. Regarding the mode of presentation to the emergency department, 52.4% arrived via ambulance (112 emergency services), while 47.6% presented directly as walk-in admissions. The mean lactate level at admission was 6.43 ± 5.57 mmol/L (median: 4.3; range: 0.60–27), and the mean lactate level at the second hour was 4.79 ± 4.39 mmol/L (median: 2.55; range: 0.40–17.8). Lactate clearance was calculated as a mean of 7.59 ± 77.43%, with a median of 2.5% (range: −361.9 to 94.4). In addition, the mean delta lactate value was 1.64 ± 5.15 mmol/L (median: 0.8; range: −9.6 to 25.5). Ethanol levels were highly variable, with a mean of 55.28 ± 105.89 mg/dL (median: 0; range: 0–397). Troponin levels generally remained within normal limits, with a mean of 0.06 ± 0.14 ng/mL (median: 0.0; range: 0–0.71). Among the acid-base parameters, the mean base excess was −17.66 ± 9.90 mmol/L (median: −17; range: −28.1 to 9.6). The glucose-to-potassium ratio, used to assess electrolyte balance, averaged 39.49 ± 27.12 (median: 35; range: 4.4–181.5). The anion gap was markedly elevated, with a mean value of 24.47 ± 9.25 mmol/L (median: 23; range: 6.9–50.2).

Regarding clinical findings, 25.6% of patients presented with visual disturbances, 43.9% with altered mental status, 20.7% with suspected alcohol intake, 3.7% with nausea and vomiting, 2.4% with syncope, and 3.7% with dyspnea. Outcome analysis revealed that 11.0% of patients died during hospitalization, 54.9% required intensive care admission, 15.9% were followed in the ward, 13.4% were discharged, and 4.9% left the hospital against medical advice.

**Fig. 2:**
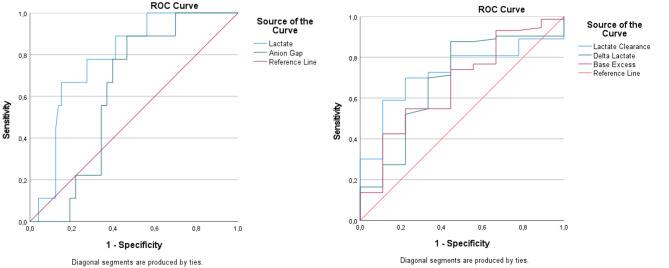
Receiver operating characteristic (ROC) curves of lactate, lactate clearance, delta lactate, base excess, and anion gap for the prediction of mortality

In patients who died, BUN (22.4 ± 17.6 vs. 11.1 ± 9.0 mg/dL), creatinine (1.58 ± 0.63 vs. 1.13 ± 0.34 mg/dL), glucose (231.7 ± 167.4 vs. 162.8 ± 101.8 mg/dL), and troponin (0.10 ± 0.19 vs. 0.03 ± 0.06 ng/mL) levels were significantly higher (all *p* < 0.05). Conversely, pH, HCO₃^−^, and base excess values were significantly lower in the non-survivor group (*p* < 0.05). Notably, lactate levels were markedly higher in non-survivors (9.04 ± 6.09 vs. 4.21 ± 3.83 mmol/L, *p* < 0.001), while lactate clearance was significantly negative (−33.4 ± 95.3 vs. 43.1 ± 29.9, *p* < 0.001). Delta lactate values also differed significantly, showing a decline in the non-survivor group (*p* < 0.001). Receiver operating characteristic (ROC) analysis demonstrated that lactate level had the highest predictive value for mortality, with a cut-off > 8.5 mmol/L (AUC = 0.785), yielding a sensitivity of 77.8% and specificity of 72.6%. For lactate clearance, a cut-off value of < 7.44 yielded an AUC of 0.725, demonstrating a meaningful predictive performance for mortality with a sensitivity of 69.9% and a specificity of 77.8%. Delta lactate (≤0.25) and base excess ( > −24.5) showed moderate discriminative ability, with AUC values of 0.683 and 0.669, respectively. The anion gap cut-off value of ≤23.3 exhibited a more limited discriminative performance (AUC = 0.626), while maintaining a high sensitivity (88.9%) (Tables 1 and 2, Fig. 2).

**Table 1:** Comparison of all variables according to mortality status

Variable	n	Exitus (Yes)	Exitus (None)	p value
Age (years) M (Min-Max)	82	60 (48–70)	56 (16–77)	0.106¥
BUN (mg/dL) M (Min-Max)	82	11 (5–73)	13 (3–94)	0.001¥
Creatinine (mg/dL) M (Min-Max)	82	1.7 (0.97–4.01)	1.24 (0.45–2.34)	0.001¥
pH Mean ± SD	82	6.94 ± 0.25	7.12 ± 0.20	0.002¢
Lactate (mmol/L) M (Min-Max)	82	11 (3.1–18)	3.7 (0.6–27)	0.001¥
Lactate2 (mmol/L) M (Min-Max)	82	10.1 (5.2–17.8)	2.2 (0.4–13.9)	0.001¥
Lactate Clearance (%) M (Min-Max)	82	−18.02 ((−78)-52.73)	38.78 ((−361.9)-94.44)	0.001¥
PCO₂ (mmHg) M (Min-Max)	82	44 (20.5–68.2)	32.7 (5.2–120)	0.355¥
Ethanol (mg/dL) M (Min-Max)	82	47 (20.5–252.9)	32.7 (5.2–397)	0.663¥
Troponin (ng/mL) M (Min-Max)	82	0.01 (0–0.71)	0.006 (0–0.71)	0.044¥
Glucose (mg/dL) M (Min-Max)	82	207 (32–302)	145 (47–949)	0.037¥
Potassium (mmol/L) M (Min-Max)	82	5.5 (3.2–7.5)	4.8 (2.6–8.4)	0.165¥
Sodium (mmol/L) Mean ± SD	82	137.54 ± 6.29	136.78 ± 4.76	0.547¢
Chloride (mmol/L) M (Min-Max)	82	102 (86–1006)	102 (83–110)	0.520¥
HCO₃ (mmol/L) M (Min-Max)	82	7.7 (4.3–22.4)	9.4 (3.8–30.9)	0.035¥
Base excess (mmol/L) M (Min-Max)	82	−24.6 ((−27.9)-(−2.2))	−20.8 ((−28.1)-9.6)	0.015¥
Glucose/Potassium Ratio M (Min-Max)	82	36.3 (4.44–55.71)	30 (8.73–181.45)	0.146¥
Anion Gap (mmol/L) Mean ± SD	82	26.92 ± 9.47	22.47 ± 8.67	0.036¢
Delta Lactate (mmol/L) M (Min-Max)	82	9.74 ((−3.9)-5.8)	0.8 ((−9.6)-25.5)	< 0.001¥

^¥^: Median(Min-Max), ^¢^: Mean ± SD

**Table 2:** ROC Curve analysis of lactate, lactate clearance, delta lactate, base excess, and anion gap values according to mortality outcomes

Variable	Cut-off	AUC	Std. Error	p value	95% CI Lower–Upper	Youden Index (J)	Sensitivity (%)	Specificity (%)
Lactate	> 8.5	0.785	0.064	**0.006**	0.659–0.910	0.504	77.78	72.6
Lactate Clearance	< 7,44	0.725	0.071	**0.029**	0.586–0.863	0,460	69,90	77,80
Delta Lactate	≤0,25	0.683	0.098	0.074	0.492–0.875	0,366	69,90	66,70
Base Excess	> −24,5	0.669	0.094	0.100	0.484–0.854	0,296	74,00	55,60
Anion Gap	≤23.3	0.626	0.067	0.221	0.495–0.756	0.423	88.89	53.42

AUC:Area Under Curve, CI: Confidence Interval

When clinical findings were compared between patients with and without mortality, **no statistically significant differences** were observed in the frequencies of **visual impairment, confusion, suspicious alcohol consumption, nausea/vomiting, syncope, or dyspnea** (*p* = 0.647). None of these presenting clinical features were significantly associated with mortality.

In contrast, **clinical endpoints differed significantly between the groups** (*p* < 0.001). All patients in the mortality group resulted in death (100%), whereas among survivors, the most common outcomes were **intensive care unit admission (86.7%)** and **ward admission (100%)**. Additionally, outcomes such as **unauthorized abandonment (100%)** and **discharge (100%)** were observed exclusively in the non-mortality group. These findings indicate a strong association between mortality status and clinical outcomes (Table 3).

**Table 3:** Comparison of all variables according to mortality status

	Exitus (Yes) n	Exitus (None) n	p value
**Clinical Findings**, *n* (%)			
Visual impairment Confusion Suspicious alcohol consumption Nausea, vomiting Syncope Dyspnea	5 (23.8) 7 (19.4) 2 (11,8) 0 (0,0) 1 (50.0) 0 (0,0)	16 (76.2) 29 (80.6) 15 (88.2) 3 (100.0) 1 (50.0) 3 (100.0)	0.647*
**Endpoint**, *n* (%)			
Ex Intensive care Service Unauthorized abandonment Discharged	9 (100,0)^*a*^ 6 (13,3)^*a*^ 0 (0,0)^*a*^ 0 (0,0)^*a*^ 0 (0,0)^*a*^	0 (0, 0)^*b*^ 39 (86 [7]), ^*a*^ 13 (100,0)^*a*^ 4 (100,0)^*a*^ 11 (100,0)^*a*^	< 0.001*

%: Percentage of rows, * Significance value obtained using the exact method

**Fig. 3:**
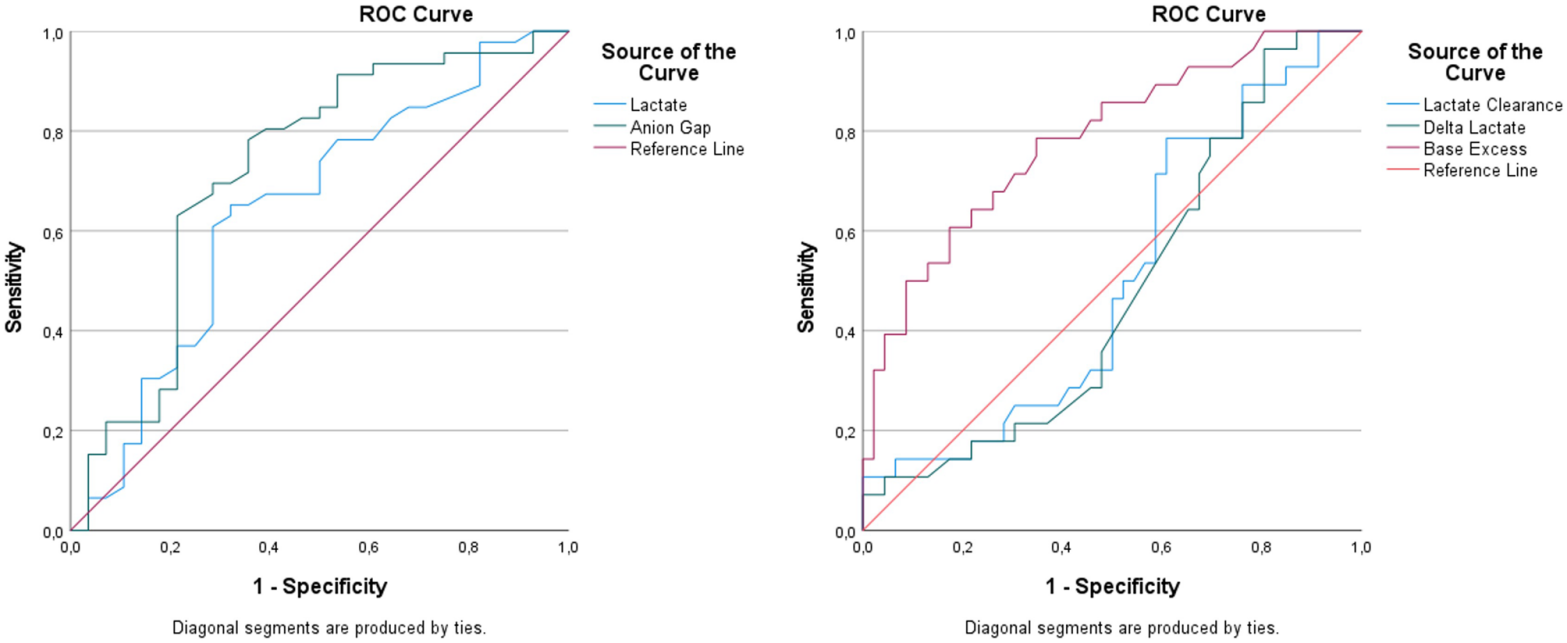
Receiver operating characteristic (ROC) curves of lactate, lactate clearance, delta lactate, base excess, and anion gap for the prediction of intensive care unit admission

In patients requiring intensive care admission, BUN (20.2 ± 16.6 vs. 9.8 ± 6.4 mg/dL) and creatinine (1.49 ± 0.61 vs. 1.09 ± 0.27 mg/dL) levels were significantly higher (*p* = 0.001). In this group, pH and HCO₃^−^ values were lower (*p* = 0.002 and *p* = 0.073, respectively). Both lactate and second-hour lactate levels were significantly elevated (*p* < 0.001), while lactate clearance was reduced (*p* = 0.001). In addition, base excess and anion gap values were also associated with the need for intensive care (*p* < 0.05). According to the ROC analysis, base excess and anion gap were identified as the parameters with the highest discriminative ability for predicting intensive care unit (ICU) admission. A cut-off value of > −21.20 for base excess yielded an AUC of 0.783, demonstrating a significant predictive performance with a sensitivity of 78.6% and a specificity of 65.2%. Similarly, the anion gap cut-off value of ≤−0.4 showed a good discriminative ability with an AUC of 0.722, providing a sensitivity of 78.3% and a specificity of 64.3%. For lactate levels, a cut-off value of > 3.6 mmol/L resulted in an AUC of 0.644, indicating a moderate discriminative ability. In contrast, lactate clearance ( < 37.08) and delta lactate (≤0.65) demonstrated limited predictive value for ICU admission, with AUC values of 0.504 and 0.480, respectively (Tables 4 and 5, Fig. 3).

**Table 4:** Comparison of all variables according to the condition of intensive care admission

Variable	n	ICU (Yes)	ICU (None)	p value
Age (years) M (Min-Max)	82	56.5 (16–77)	54.5 (19–74)	0.119¥
BUN (mg/dL) M (Min-Max)	82	14 (5–94)	11.5 (3–73)	0.001¥
Creatinine (mg/dL) M (Min-Max)	82	1.35 (0.73–2.87)	1.01 (0.45–4.01)	0.001¥
pH Mean ± SD	82	6.98 ± 0.23	7.15 ± 0.19	0.002¢
Lactate (mmol/L) M (Min-Max)	82	6.15 (1–22)	3 (0.6–27)	0.001¥
Lactate2 (mmol/L) M (Min-Max)	82	3.65 (0.4–17.8)	1.8 (0.4–14.3)	0.001¥
Lactate Clearance (%) M (Min-Max)	82	37.24 ((−361.9)-78.26)	30 ((−133.33)-94.44)	0.001¥
PCO₂ (mmHg) M (Min-Max)	82	28.5 (5.2–58.7)	39.95 (11.5–120)	0.555¥
Ethanol (mg/dL) M (Min-Max)	82	28.5 (5.2–397)	61.15 (11.5–347.6)	0.583¥
Troponin (ng/mL) M (Min-Max)	82	0.006 (0–0.71)	0.01 (0–0.71)	0.066¥
Glucose (mg/dL) M (Min-Max)	82	195 (48–949)	113 (32–400)	0.151¥
Potassium (mmol/L) M (Min-Max)	82	5.3 (2.6–8.4)	4.04 (3.1–7.2)	0.146¥
Sodium (mmol/L) Mean ± SD	82	137.43 ± 6.03	136.68 ± 4.94	0.573¢
Chloride (mmol/L) M (Min-Max)	82	102 (83–110)	104 (86–109)	0.444¥
HCO₃ (mmol/L) M (Min-Max)	82	7.55 (3.8–23.2)	12.2 (3.8–30.9)	0.073¥
Base excess (mmol/L) M (Min-Max)	82	−23.6 ((−20.1)-0.9)	−15.45 ((−27.10)-9.6)	0.026¥
Glucose/Potassium Ratio M (Min-Max)	82	34.31 (8.73–181.45)	29.61 (4.44–100)	0.271¥
Anion Gap (mmol/L) M (Min-Max)	82	26 (9.8–44.6)	20.45 (6.9–50.2)	0.039¥
Delta Lactate (mmol/L) M (Min-Max)	82	0.9 ((−9.6)-14.6)	0.6 ((−4.7)-25.5)	0.001¥

¥:Median(Min-Max), ¢:Mean ± SD

**Table 5:** ROC Curve analysis of lactate, lactate clearance, delta lactate, base excess, and anion gap values based on intensive care unit admission

Variable	Cut-off	AUC	Std. Error	p value	95% CI Lower–Upper	Youden Index (J)	Sensitivity (%)	Specificity (%)
Lactate	> 3.6	0.644	0.069	0.039	0.509–0.778	0.331	65.22	67.86
Lactate Clearance	< 37,08	0,504	0.069	0.956	0,368–0639	−0,030	50,00	47,80
Delta Lactate	≤0,65	0,480	0.068	0.776	0,346–0614	−0,065	50,00	43,50
Base Excess	> −21,20	0,783	0.055	**0.001**	0,674–0891	0,438	78,60	65,20
Anion Gap	≤ − 0.4	0.722	0.066	**0.001**	0.594–0.851	0.425	78.26	64.29

AUC:Area Under Curve, CI: Confidence Interval

**Fig. 4:**
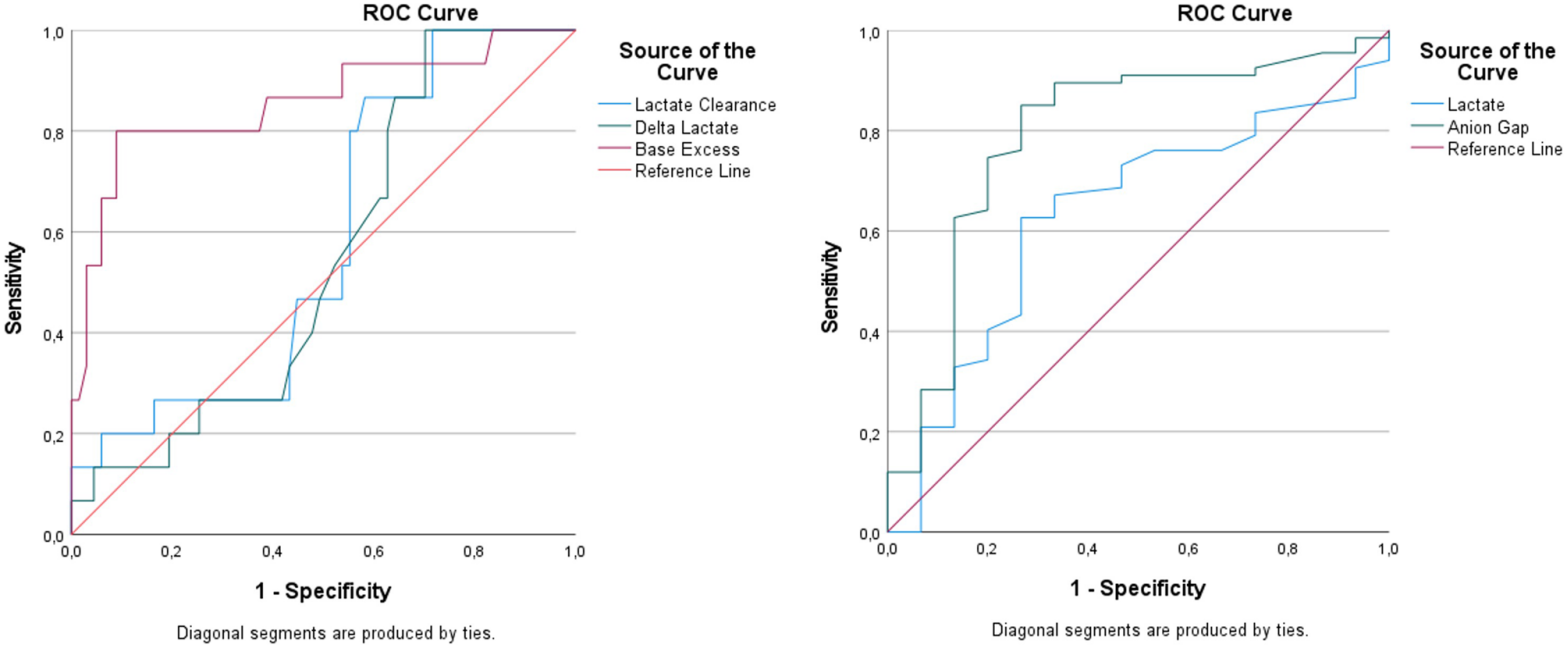
Receiver operating characteristic (ROC) curves of lactate, lactate clearance, delta lactate, base excess, and anion gap for the prediction of discharge

In patients who were discharged, BUN levels were significantly lower (10.1 ± 6.1 vs. 18.7 ± 15.8 mg/dL, *p* = 0.017), while pH values were higher (7.15 ± 0.14 vs. 7.02 ± 0.23, *p* = 0.038). Lactate and second-hour lactate levels were also significantly lower in this group (*p* = 0.008 and *p* = 0.004, respectively). In addition, lactate clearance was higher (47.1 ± 32.1 vs. −2.3 ± 76.3, *p* = 0.007), and delta lactate values trended positively (*p* = 0.015). ROC curve evaluation identified base excess and anion gap as the parameters with the strongest discriminative performance for predicting discharge. A cut-off value of > −8.05 for base excess yielded an AUC of 0.856, demonstrating an excellent predictive performance with a sensitivity of 80.0% and a specificity of 91.0%. The anion gap cut-off value of ≤18.4 also showed good discriminative ability, with an AUC of 0.793, providing a sensitivity of 85.1% and a specificity of 73.3%.For lactate levels, a cut-off value of > 3.6 mmol/L resulted in an AUC of 0.632, indicating a limited to moderate discriminative ability for discharge. In contrast, lactate clearance ( < 33.13) and delta lactate ( < 0.55) did not demonstrate meaningful discriminative performance for predicting discharge, with AUC values of 0.581 and 0.547, respectively (Tables 6 and 7, Fig. 4).

**Table 6:** Comparison of all variables according to discharge status

Variable	n	Discharge (Yes)	Not Discharged	p value
Age (years) M (Min-Max)	82	50 (19–66)	57 (16–77)	0.772¥
BUN (mg/dL) M (Min-Max)	82	11 (3–26)	13 (5–94)	0.017¥
Creatinine (mg/dL) M (Min-Max)	82	0.87 (0.65–1.58)	1.31 (0.45–4.01)	0.059¥
pH M (Min-Max)	82	7.3 (6.9–7.5)	7 (6.55–7.41)	0.038¥
Lactate (mmol/L) M (Min-Max)	82	2.1 (1.3–27)	5 (0.6–22)	0.008¥
Lactate2 (mmol/L) M (Min-Max)	82	1.8 (0.4–5.2)	3.3 (0.4–17.8)	0.004¥
Lactate Clearance (%) M (Min-Max)	82	33.3 ((−16.67)-94.44)	35.29 ((−361.9)-89.41)	0.007¥
PCO₂ (mmHg) M (Min-Max)	82	41.8 (12.9–54.1)	30.9 (5.2–120)	0.536¥
Ethanol (mg/dL) M (Min-Max)	82	105.8 (12.9–346)	30.9 (5.2–397)	0.175¥
Troponin (ng/mL) M (Min-Max)	82	0.03 (0–0.54)	0.007 (0–0.71)	0.232¥
Glucose (mg/dL) M (Min-Max)	82	103 (80–222)	184 (32–949)	0.244¥
Potassium (mmol/L) M (Min-Max)	82	3.8 (3.1–6.1)	5.23 (2.6–8.4)	0.197¥
Sodium (mmol/L) Mean ± SD	82	136.00 ± 4.71	137.34 ± 5.89	0.397¢
Chloride (mmol/L) M (Min-Max)	82	104 (94–109)	102 (83–110)	0.555¥
HCO₃ (mmol/L) M (Min-Max)	82	22.7 (5–30.9)	7.9 (3.8–23.4)	0.095¥
Base excess (mmol/L) M (Min-Max)	82	−1.1 ((−26.6)-9.6)	−22.2 ((−28.1)-0.9)	0.191¥
Glucose/Potassium Ratio M (Min-Max)	82	28.61 (15.08–47.89)	32.55 (4.44–181.45)	0.529¥
Anion Gap (mmol/L) M (Min-Max)	82	13.3 (7.7–37.3)	25.7 (6.9–50.2)	0.251¥
Delta Lactate (mmol/L) M (Min-Max)	82	0.7 ((−0.3)-25.5)	0.7 ((−9.6)-15.2)	0.015¥

^¥^: Median(Min-Max), ^¢^: Mean ± SD

**Table 7:** ROC curve analysis of lactate, lactate clearance, delta lactate, base excess, and anion gap values based on discharge outcome

Variable	Cut-off	AUC	Std. Error	p value	95% CI Lower–Upper	Youden Index (J)	Sensitivity (%)	Specificity (%)
Lactate	> 3.6	0.632	0.078	0.112	0.480–0.784	0.360	62.69	73.33
Lactate Clearance	< 33,13	0,581	0.074	0.331	0,435–0726	−0,004	53,30	46,30
Delta Lactate	< 0,55	0,547	0.072	0.569	0,406–0689	0,330	60,00	43,30
Base Excess	> −8,05	0,856	0.064	**0.001**	0,731–0981	0,710	80,00	91,00
Anion Gap	≤18.4	0.793	0.070	**0.001**	0.655–0.931	0.584	85.07	73.33

AUC: Area Under Curve, CI: Confidence Interval

## Dıscussıon

Methanol intoxication is a toxicological condition with high mortality and morbidity, characterized by a rapidly progressive clinical course in which early intervention is crucial. Therefore, identifying reliable biomarkers to predict mortality is essential for optimizing patient management in emergency departments [1].

Analysis of demographic data revealed that the mean age was significantly higher in the non-survivor group. Previous studies have also reported that advanced age increases the risk of mortality in methanol intoxication [15, 16]. This may be attributed to age-related decline in renal function and cardiovascular reserve, which reduces the ability to tolerate severe metabolic acidosis and hypoxia. In addition, the higher prevalence of comorbidities and diminished physiological reserves in elderly patients may hinder the elimination of toxic metabolites, thereby worsening prognosis. Our findings indicate that elderly patients may require closer monitoring and more aggressive therapeutic strategies.

Evaluation of laboratory parameters showed significantly higher creatinine levels in the non-survivor group. This suggests that insufficient renal elimination of toxic metabolites may contribute to mortality. Impaired renal clearance can accelerate the accumulation of formic acid, leading to deeper metabolic acidosis and more severe tissue hypoxia. Previous studies have also emphasized the prognostic role of renal dysfunction in methanol intoxication [17]. Furthermore, lower HCO₃^−^ levels and markedly acidotic pH values strongly support the association between severe metabolic acidosis and mortality, while elevated PCO₂ values indicate inadequate respiratory compensation. This implies that the respiratory burden fails to correct metabolic imbalance, further compromising oxygen delivery and increasing mortality risk [18]. To date, there are no studies in the literature directly addressing the impact of acute methanol ingestion on cardiac injury. Elevated troponin levels in this study suggest that metabolic acidosis rapidly induces multi-organ damage, including cardiac involvement, and may represent an important predictor of mortality. Collectively, these findings demonstrate that close monitoring of renal and cardiac function, together with careful evaluation of acid–base balance, plays a vital role not only in predicting mortality but also in guiding therapeutic strategies in the management of methanol intoxication.

The most critical finding in this study was the dynamics of lactate. In patients who died, lactate levels were significantly elevated, while lactate clearance was markedly reduced. This suggests that a single baseline lactate measurement is not sufficient, and that changes in lactate levels during treatment play a crucial role in prognostic assessment. Although delta lactate alone was found to be statistically significant as a predictor of mortality, its inability to provide a meaningful cut-off value indicates that, compared with lactate or lactate clearance alone, its use may be less practical. This finding implies that complex metabolic processes superimposed on the patient’s condition may contribute to variability in delta lactate values.

In methanol metabolism, inhibition of the cytochrome C oxidase enzyme by formic acid halts cellular energy production despite the presence of oxygen, resulting in histotoxic hypoxia. This process disrupts mitochondrial oxidative phosphorylation and shifts metabolism toward anaerobic pathways, thereby increasing lactate production. The reduced lactate clearance observed in our findings demonstrates that this mitochondrial blockade cannot be fully corrected despite treatment, leading to persistent tissue hypoperfusion. This highlights that the hypoxic process may continue despite clinical management, biochemical recovery may lag behind clinical stabilization, and mortality risk may persist. Similar findings have been consistently reported in the literature, where high lactate clearance is strongly associated with survival, while low clearance is indicative of treatment failure and poor outcomes in conditions such as sepsis and cardiac arrest [19–21]. Our results therefore suggest that lactate clearance is a reliable parameter for monitoring treatment response and identifying high-risk patients in methanol intoxication. This study demonstrates that both baseline lactate measurement and lactate clearance can serve as valuable tools for early risk stratification in predicting mortality. For example, a recent study has shown that lactate-to-albumin ratio (LAR) and albumin-corrected anion gap are significant prognostic markers in methanol poisoning. This finding is consistent with our study’s conclusion that anion gap has predictive power for ICU admission and discharge [22, 23].

Patients with elevated baseline lactate levels but rapid subsequent clinical improvement may be misclassified as high-risk when assessment relies solely on a single measurement. In our cohort, however, the AUC value calculated for lactate clearance demonstrated lower discriminatory power than that of lactate itself. This finding suggests that lactate values reflect not only the initial metabolic disturbance but also therapeutic response and improvements in tissue perfusion. In line with prior studies, Büberci et al. [24] identified a threshold of 5.75 mmol/L in 18 patients, while Kayalı et al. [25] reported 4.35 mmol/L in a cohort of 49 patients, both significantly associated with increased mortality. In our study, based on 82 patients, the threshold was higher at > 8.5 mmol/L. Other investigations have also demonstrated that lactate is strongly correlated with mortality [26], whereas lactate clearance has been associated with methanol levels [27]. Consistent with these reports, our findings confirm that elevated lactate levels and reduced lactate clearance are robust predictors of mortality risk [28]. The literature also contains studies showing that lactate’s prognostic power increases when used not alone, but in ratio with other parameters such as hemoglobin or albumin, or by calculating its clearance [29, 30]. For example, lactate clearance and lactate-hemoglobin ratio have been reported as strong mortality predictors in critically ill patients presenting to the emergency department [29]. This finding supports the prognostic superiority of lactate and lactate clearance in our study.

When considering outcomes such as hospital discharge and intensive care admission, troponin—a marker of cardiac toxicity—did not reach statistical significance. Although lactate, lactate clearance, and delta lactate were individually significant, ROC analysis revealed that base excess and anion gap provided more reliable cut-off values for predicting both ICU admission and discharge. This represents a distinctive and noteworthy aspect of our study. It is also well established that the anion gap correlates with serum formate concentrations [31].

Although base excess and anion gap have both been reported as significantly elevated in non-survivors, our study demonstrated that thresholds of ≤18.4 mmol/L for anion gap and >–28.1 mmol/L for base excess predicted discharge, whereas ≤–0.4 mmol/L and > 10 mmol/L, respectively, predicted ICU admission. To the best of our knowledge, no prior studies have identified predictive roles for base excess or anion gap in relation to early discharge among patients with methanol intoxication. These findings indicate that these parameters may serve as valuable, accessible tools in the emergency department, supporting early decisions regarding antidote administration or urgent hemodialysis. Base excess and anion gap were associated with intensive care unit admission and hospital discharge, but these are clinical management decisions, not biological endpoints. ICU admission depends on physician judgment, institutional protocols, and resource availability, while discharge decisions consider clinical stability, laboratory improvement, and treatment needs. Thus, associations between metabolic severity and disposition decisions should be interpreted as correlations rather than causal predictors of clinical outcomes.

Glucose is considered an important biomarker for predicting early mortality in methanol poisoning [32]. Although the direct prognostic value of potassium is limited, overall electrolyte imbalances and the severity of acidosis play a critical role in determining disease progression [33]. In this study, it was hypothesized that the glucose/potassium ratio might be significant in predicting mortality and the need for intensive care admission. However, in line with the literature, glucose was found to be significant only in predicting mortality, whereas the glucose/potassium ratio at initial admission to the emergency department did not provide meaningful prognostic information. This finding suggests that patients presented to the emergency department at an early stage of intoxication or were in the initial phase of renal injury.

In addition, this study revealed that clinical symptoms (such as visual disturbances, altered mental status, nausea-vomiting, etc.) were not significantly associated with mortality. This may be explained by the variability in the onset of symptoms depending on the amount of methanol ingested, duration of exposure, and individual metabolic differences. Indeed, Jangyou et al. reported that clinical manifestations in methanol intoxication are generally nonspecific and unreliable for predicting early mortality [2]. Furthermore, although the accumulation of formic acid exerts toxic effects on the optic nerve and central nervous system, leading to findings such as vision loss and altered consciousness, these symptoms are not directly correlated with mortality [34]. These findings indicate that risk stratification based solely on clinical symptoms may be misleading and highlight the importance of laboratory parameters that provide more objective data in predicting mortality.

### Limitations

This study has several limitations that should be acknowledged. The retrospective design limits the ability to establish causal relationships and increases the potential for missing data. The relatively small sample size and low mortality rate may have reduced the statistical power of the analysis; therefore, multivariable regression analyses were not performed, and the findings were interpreted cautiously within the framework of descriptive and univariable analyses. Moreover precise timing of therapeutic interventions relative to serial lactate measurements could not be standardized or analyzed. Consequently, lactate clearance should not be interpreted as a direct measure of treatment efficacy, but rather as an integrated marker of early metabolic trajectory and disease severity. As the study was conducted at a single center, the generalizability of the results to other populations may be limited. Lactate measurements were obtained only at predefined time points, which may not fully capture dynamic changes throughout the clinical course. The identified base excess and anion gap cut-off values should be considered exploratory and hypothesis-generating, requiring external validation in prospective and multicenter cohorts before clinical application. In addition, because serum methanol levels are not routinely measurable at our center, the diagnosis was established based on a combination of clinical findings, patient history, and laboratory parameters. While this approach reflects real-world clinical practice, the lack of biochemical confirmation should be considered an additional limitation of the study.

## Conclusion

This study shows that both lactate levels and lactate clearance are strong prognostic indicators in methanol intoxication. Dynamic monitoring of lactate provides more reliable information than a single measurement, with low clearance associated with poor outcomes. Additionally, base excess and anion gap at admission may help predict intensive care needs and discharge potential. Incorporating these parameters into routine emergency assessment could enhance early risk stratification and patient management.

## Data Availability

The datasets generated and analyzed during the current study are available from the corresponding author on reasonable request.
